# Investigation of measurable residual disease in acute myeloid leukemia by DNA methylation patterns

**DOI:** 10.1038/s41375-021-01316-z

**Published:** 2021-06-15

**Authors:** Tanja Božić, Chao-Chung Kuo, Jan Hapala, Julia Franzen, Monika Eipel, Uwe Platzbecker, Martin Kirschner, Fabian Beier, Edgar Jost, Christian Thiede, Wolfgang Wagner

**Affiliations:** 1 Helmholtz-Institute for Biomedical Engineering, Stem Cell Biology and Cellular Engineering, RWTH Aachen University Medical School, Aachen, Germany; 2 Institute for Biomedical Engineering – Cell Biology, RWTH Aachen University Medical School, Aachen, Germany; 3 Department of Hematology, Cellular Therapy and Hemostaseology, Leipzig University Hospital, Leipzig, Germany; 4 Department of Hematology, Oncology, Hemostaseology and Stem Cell Transplantation, Medical School, RWTH Aachen University, Aachen, Germany; 5 Center for Integrated Oncology Aachen Bonn Cologne Düsseldorf (CIO ABCD), Aachen, Germany; 6 Medical Department I, University Hospital Carl Gustav Carus, TU Dresden, Dresden, Germany

**Keywords:** Cancer epigenetics, Acute myeloid leukaemia, Cancer genomics

## Abstract

Assessment of measurable residual disease (MRD) upon treatment of acute myeloid leukemia (AML) remains challenging. It is usually addressed by highly sensitive PCR- or sequencing-based screening of specific mutations, or by multiparametric flow cytometry. However, not all patients have suitable mutations and heterogeneity of surface markers hampers standardization in clinical routine. In this study, we propose an alternative approach to estimate MRD based on AML-associated DNA methylation (DNAm) patterns. We identified four CG dinucleotides (CpGs) that commonly reveal aberrant DNAm in AML and their combination could reliably discern healthy and AML samples. Interestingly, bisulfite amplicon sequencing demonstrated that aberrant DNAm patterns were symmetric on both alleles, indicating that there is epigenetic crosstalk between homologous chromosomes. We trained shallow-learning and deep-learning algorithms to identify anomalous DNAm patterns. The method was then tested on follow-up samples with and without MRD. Notably, even samples that were classified as MRD negative often revealed higher anomaly ratios than healthy controls, which may reflect clonal hematopoiesis. Our results demonstrate that targeted DNAm analysis facilitates reliable discrimination of malignant and healthy samples. However, since healthy samples also comprise few abnormal-classified DNAm reads the approach does not yet reliably discriminate MRD positive and negative samples.

## Introduction

Many patients with acute myeloid leukemia (AML) achieve hematologic remission after treatment, but most of them will eventually experience relapse [1]. Early and accurate detection of remaining leukemia cells—previously referred to as “minimal residual disease” (MRD), and now more appropriately termed “measurable residual disease” with the same acronym—has been shown to be a strong prognostic indicator for relapse of AML, and might therefore be used as parameter for deciding on post-remission treatment [2, 3].

Assessment of MRD is based on detection of specific immunophenotypic [4], or genotypic [5] aberrations. This is commonly performed by multiparameter flow cytometry (MFC) of leukemia-associated immunophenotypes, and detection of specific mutations by real-time quantitative PCR (RT-qPCR), digital droplet PCR, or next generation sequencing (NGS) [1, 3]. So far, RT-qPCR is considered as a gold standard for detection of MRD, however, it is applicable to only 40–60% of AML patients as not all patients have suitable translocations or mutations [3, 6]. Frameshift mutations of nucleophosmin 1 (*NPM1*) are one of the most frequent molecular abnormalities in AML that remain relatively stable in the course of the disease and therefore represent an ideal target for RT-qPCR MRD monitoring [7, 8]. Immunophenotypic detection of MRD can potentially investigate more than 85% of AML cases, but this approach lacks standardization of selected markers, instrument settings or reporting thresholds [9].

Aberrant DNA methylation (DNAm) is a hallmark of cancer, which is generally observed in AML [10, 11]. It is characterized by a genome-wide loss of DNAm and accompanying hypermethylation of promoter-associated CpG-islands [12]. The epigenetic landscape of AML is particularly modified by mutations and epimutations in epigenetic modifiers, such as DNA methyltransferase *DNMT3A* [13–15] and *TET2* [16]. Several studies demonstrated that DNAm can be utilized as a biomarker for AML diagnosis, classification, and prognostic stratification [17–19]. For example, AML-subtypes reveal specific genome-wide DNAm signatures [20, 21].

In this study, we followed the hypothesis that DNAm can also be used to estimate MRD based on residual abnormal epigenetic patterns. To this end, we identified genomic regions, which generally reveal aberrant DNAm in AML. Targeted analysis of these regions by bisulfite amplicon sequencing (BA-seq) with shallow-learning and deep-learning algorithms, was then used to stratify DNAm patterns of individual reads as either normal or abnormal.

## Materials and methods

### Identification of AML-associated CpGs

To identify CpGs that generally reveal aberrant DNAm in AML we used Illumina Human Methylation 450k BeadChip datasets of blood from healthy donors: GSE40279 [22], GSE50660 [23] and GSE77716 [24]; and from bone marrow of AML patients: The Cancer Genome Atlas (TCGA) [25], GSE58477 (cytogenetic normal AMLs) [26] and GSE62298 [27]. To identify reliable and reproducible candidate CpGs we randomly paired these control and AML datasets. For each of these paired studies the candidate CpGs were selected based on: (i) *β*-value (DNAm level) < 0.1 or >0.9 in healthy donors; (ii) standard deviation (s.d.) of DNAm in healthy samples <0.05; (iii) and then ranked by the mean DNAm difference between healthy and AML in the corresponding datasets. Based on these criteria, we selected the top 100 CpGs that were either hyper- or hypomethylated in AML. The DNAm of selected CpGs was also analyzed in DNAm datasets (all Human Methylation 450k BeadChip) of leukocyte subsets (GSE35069) [28]; hematopoietic stem and progenitor cells (GSE63409) [29]; CD34 ^+^ cells (GSE58477) [26], B-cell lymphoma (GSE37362) [30]; myelodysplastic syndrome (MDS; GSE51758) [31]; AML of elderly (GSE86409) [32], IDH1/IDH2-mutant AML (GSE153347), AML1-ETO (GSE80508), FLT3-ITD (GSE64934) [33], and MDS and secondary AML (GSE152710) [34].

### Collection and preparation of samples

For targeted analysis of DNAm we used 34 blood samples of 15 AML patients (first diagnosis and follow-up samples) from the Department of Hematology, Oncology, Hemostaseology, and Stem Cell Transplantation; 83 blood samples of 34 AML patients (first diagnosis and one or more follow-up samples) obtained from the Study Alliance Leukemia Biobank (SAL, Dresden, Germany); and peripheral blood of 63 healthy donors from the Department of Transfusion Medicine at RWTH Aachen University Medical School. The study was approved and all samples were taken after written consent according to the guidelines of the local ethics committees of RWTH Aachen University (Permit Numbers: EK206/09 (AML) and EK099/14 (healthy donors)) and of the Medical Faculty of the Technical University of Dresden (EK98032010). All AML samples at first diagnosis of the SAL Biobank carry a mutation in *NPM1* and MRD monitoring was performed based on RT-qPCR using locked nucleic acid-containing primers adjusted to *ABL1* as a reference gene (*NPM1*^mut^/ABL) as described previously [35]. Genomic DNA was isolated from blood samples with the QIAamp DNA Blood Kit (Qiagen, Hilden, Germany).

To estimate the sensitivity of our DNAm-based approach we performed a limiting dilution assay by mixing DNA of a control sample (sample ID: DD_H1_100) with DNA of an AML sample exhibiting high blast count in the bone marrow and peripheral blood. Furthermore, we analyzed the DNAm pattern of six leukemia cell lines: HL-60 (AML), KG-1a (AML), TF-1 (AML), K562 (chronic myeloid leukemia), U937 (histiocytic lymphoma) and SUP-B15 (B-cell precursor leukemia). DNA was isolated with the NucleoSpin Tissue Kit (Macherey-Nagel, Düren, Germany). Further information on AML samples is provided in Supplemental Table S1.

### Bisulfite amplicon sequencing

Assays for targeted BA-seq were designed for four regions with AML-associated CpGs. They comprise 14 neighboring CpGs around cg15289427, 10 CpGs around cg22797031, 15 CpGs around cg27630153, and 9 CpGs around cg19586199. Genomic DNA (500 ng) of blood samples or cell lines was bisulfite converted with the EZ DNAm (Zymo Research, Irvine, USA). Subsequently, the four AML-associated regions were amplified using the PyroMark PCR Kit (Qiagen) using primers with overhangs for subsequent barcoding (Supplemental Table S2). PCR conditions are provided in the Supplemental Table S3. Subsequently, all four regions of a sample were pooled and purified using the Agencourt AMPure XP PCR Purification Kit (Beckman Coulter, Brea, CA, USA). Pooled regions were amplified with a 2nd round of PCR using the PyroMark PCR Kit and primers containing 8 bp long barcodes (Supplemental Table S4). Next, all samples were pooled, purified with the Select-a-Size DNA Clean & Concentrator Kit (Zymo Research), and library concentration was measured with the Qubit dsDNA BR Assay Kit (Thermo Fischer Scientific). The prepared amplicon library was denatured and diluted using the MiSeq Reagent Nano Kit v2 or MiSeq Reagent Kit v2 (Illumina, San Diego, CA, USA). Due to low library diversity, 15–20% of the PhiX Control (Illumina) was spiked-in.

Pooled and barcoded samples were analyzed on the Illumina MiSeq Benchtop Sequencer (Illumina) using the 250 bp paired-end sequencing mode. Raw data quality was assessed with FastQC and trimmed with Trim Galore (https://www.bioinformatics.babraham.ac.uk/projects/trim_galore/). Trimmed reads were then aligned to given reference sequences and the DNAm status of each CpGs was identified for each read by Bismark [36]. The average read depth was 8,500 (cg15289427), 36,000 (cg22797031), 37,000 (cg27630153), and 51,000 (cg19586199), while all samples with <50 reads on one of the amplicons were ignored. The analysis was done in R and Python. Raw data has been deposited at Gene Expression Omnibus under the accession number GSE166264. *(Reviewer access token for GSE166264: ajyjeaqujdkfpil)*

SNP-analysis was done through splitting the reads within a sample into two groups by the base with around 50% frequency of the top two variants. This base was at least 3 bp apart from any CpG to exclude impact by DNAm. Only few patients have such SNPs in the target regions.

### AML-score based on four CpGs

The AML-score provides a simple measure based on the mean DNAm levels at the four AML-associated CpGs, resulting in a score ranging from 0 to 1.

$${\mathrm{AML}}{\hbox{-}}{\mathrm{{score}}} = \frac{{\beta _{{\mathrm{cg}}15289427} + \beta _{{\mathrm{cg}}22797031} + (1 - \beta _{{\mathrm{cg}}27630153}) + (1 - \beta _{{\mathrm{cg}}19586199})}}{4}$$

### Computational prediction of anomalous DNAm patterns

Control samples were randomly split into training and validation datasets with a 7 to 3 ratio. Normal and abnormal DNAm patterns in individual reads of BA-seq data were defined based on their occurrence in the training set of healthy donors. We used two machine learning approaches to define abnormal DNAm patterns: random forest and autoencoder. Abnormal DNAm patterns were called by the machine learning algorithm by supervised training on healthy and AML samples.

The random forest algorithm was applied with Python library scikit-learn on each AML-associated region separately. The training datasets from control samples plus additional first diagnosis samples were used to train the model with maximal tree depth of 5. Based on the classification of reads as abnormal and normal an anomaly ratio was calculated for each region. The anomaly score for a given sample is then provided as the mean of four anomaly ratios.

The autoencoder algorithm was constructed by Python library Keras with the architecture of 8-3-8 nodes for encoder, latent, and decoder layer, respectively. The binary signals for DNAm were converted to −1 (non-methylated) and 1 (methylated) as the hyperbolic tangent activation function was applied. Since we assume that DNAm patterns of different blood cells are heterogeneous, all reads of a sample were classified before they were applied for autoencoder: The classification is carried out by K-means algorithm for unsupervised classification of the distinct blood cell clusters of reads. This resulted in five clusters for each AML-associated region. The purpose was to distinguish different reads that might derive from distinct cell types, such as myeloid and lymphoid cells. The training of the autoencoder was done for each cluster on each AML-associated region. The loss function was defined by mean squared error, and the threshold for abnormal reads was defined by the 99% percentile of loss function of all reads in the training dataset. Any read above that threshold is regarded as an abnormal read. The anomaly-ratio was calculated from the proportion of detected abnormal reads, and eventually the anomaly score was calculated from the mean of anomaly-ratios of all four AML-associated regions. The anomaly score cutoffs for random forest and autoencoder were defined by 99% percentile of the control training datasets.

### Analysis of clonal hematopoiesis of indeterminate potential (CHIP)

Library preparation and sequencing (250 bp paired-end) was performed on the MiSeq Illumina platform with the MiSeq Reagent Kit V3 (MDS/MPN panel). Raw data was analyzed with Ilumina RTA software (version 1.18.54), and the SeqNEXT software (version 4.4.0, JSI medical systems GmbH, Ettenheim, Germany) was used for alignment and variant calling. The self-customized panel contained either the entire coding sequence or hot spot regions of 31 genes (*ABL1, ASXL1, BARD1, CALR, CBL, CHEK2, CSF3R, DNMT3A, ETNK1, ETV6, EZH2, IDH1, IDH2, JAK2, KIT, KRAS, MPL, NFE2, NRAS, PDGFRA, PTPN11, RUNX1, SETBP1, SF3A1, SF3B1, SH2B3* (*LNK*), *SRSF2, TCF12, TET2, TP53, U2AF1*). To minimize risk of detecting sequencing errors, a threshold of 10 (absolute) and 5% (relative) variant reads for calling a variant was chosen. After alignment, germline variants according to dbSNP(150) database were excluded and the variant list was reviewed manually.

## Results

### Specific genomic regions reveal frequent and marked aberrant DNAm in AML

**Fig. 1:**
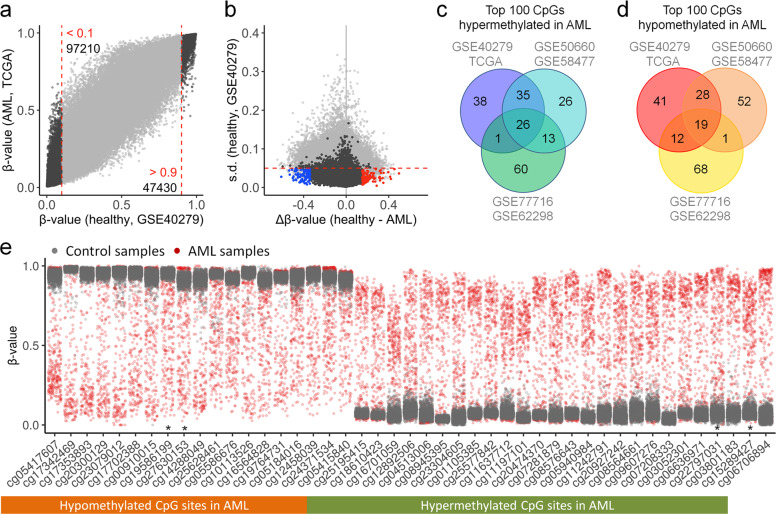
Selection of AML-associated CpGs. **a** Scatter plot of mean DNAm levels (*β*-values) in Illumina Human Methylation 450k BeadChip data of AML (TCGA; *n* = 194) *versus* normal blood samples (GSE40279; *n* = 656). The numbers of CpGs, which were consistently either non-methylated (*β*-values < 0.1) or methylated (*β*-values > 0.9) are indicated. **b** These CpGs were further filtered by low variation in controls (standard deviation [s.d.] < 0.05, red dashed line). Subsequently, the top 100 CpGs with either highest hypermethylation (blue) or hypomethylation (red) in AML were selected. **c, d** In analogy, candidate CpGs were alternatively selected with two independent pairs of control/AML studies (GSE50660/GSE58477 and GSE77716/GSE62298). Venn diagrams demonstrated the overlap of the top 100 hypermethylated (**c**) and hypomethylated CpGs in AML (**d**). **e** The distribution of DNAm levels across control (GSE40279, gray) and AML samples (TCGA, red) is depicted for each of the overlapping 19 hypo- and 26 hypermethylated CpGs (* = CpGs selected for subsequent targeted analysis).

To select genomic regions that facilitate reliable discrimination of DNAm patterns of malignant and healthy samples, we focused on CpGs that were either consistently non-methylated or methylated in healthy controls (*β*-value < 0.1 or >0.9) and had a low variation of DNAm levels in control samples (standard deviation [s.d.] < 0.05), to make sure that normal DNAm could be robustly discerned. Subsequently, we selected CpGs with the highest difference in mean DNAm between control and AML samples. This analysis was performed independently for three combinations of DNAm datasets of control *versus* AML studies: GSE40279 (*n* = 656) [22] *versus* TCGA (*n* = 194) [25]; GSE50660 (*n* = 464) [23] *versus* GSE58477 (*n* = 62) [26]; and GSE77716 (*n* = 573) [24] *versus* GSE62298 (*n* = 68) [27] (Fig. 1a,b; Supplemental Fig. S1). Notably, despite the very different studies of AML and control samples, there was a remarkable overlap in the top 100 selected CpGs: All three comparisons revealed consistent aberrant hypermethylation in AML at 26 CpGs, and hypomethylation at 19 CpGs (Fig. 1c,d; Supplemental Table S5).

When we analyzed DNAm levels at these CpGs in individual AML samples, we observed that aberrant DNAm was frequently modified to extreme opposite to the controls—often the difference of mean DNAm in control *versus* AML (Δ*β*-value) was higher than 50% (Fig. 1e). This was unexpected given that the two alleles within an AML cell are not necessarily coherently modified and that individual AML samples comprise both leukemic and normal blood cells. To further investigate if Δ*β*-values correlated with blast counts, we have compared aberrant DNAm at the 26 and 19 CpGs with blasts from AML samples of the TCGA dataset (Supplemental Fig. S2). Even AML samples with <50% bone marrow blasts showed CpGs with Δ*β*-values > 0.7 or < −0.7, indicating that the percentage of bone marrow cells with aberrant DNAm is higher than anticipated by blast counts. Taken together, we identified candidate CpGs that revealed aberrant DNAm across many AML samples and the Δ*β*-values at these sites was often remarkably high.

### Targeted analysis of CpGs with aberrant DNAm in AML

**Fig. 2:**
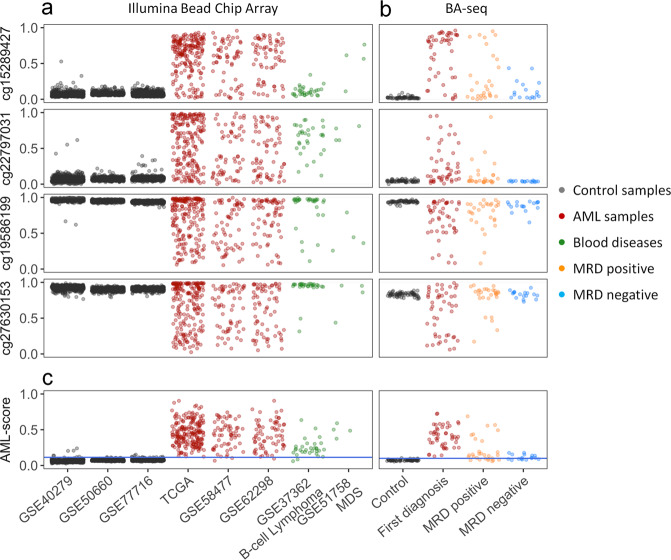
Targeted analysis of AML-associated CpGs. **a** The DNAm levels at four AML-associated CpGs (cg15289427, cg22797031, cg19586199, and cg27630153) were analyzed in Illumina Human Methylation 450k BeadChip datasets of controls, AML, and other hematologic malignancies. **b** Alternatively, DNAm was analyzed with bisulfite amplicon sequencing (BA-seq) in controls (*n* = 62), AML at first diagnosis (*n* = 48), and follow-up samples that were either classified MRD positive (*n* = 28) or MRD negative (*n* = 17) based on *NPM1*^mut^. **c** DNAm at the four AML-associated CpGs is combined into an AML-score. The cutoff of 99.5% percentile of the healthy samples is shown by the blue line.

To further curtail AML-associated regions for targeted analysis, we selected four CpGs with perturbation in most AML samples, which were associated with the genes for RHO family interacting cell polarization regulator 2 (*FAM65B*; cg15289427), piezo type mechanosensitive ion channel component 1 (*FAM38A*; cg27630153), and protein kinase CAMP-activated catalytic subunit alpha (*PRKACA*; cg19586199), while cg22797031 is not associated with a specific gene. Either way, analysis of the TCGA datasets revealed that DNAm did not correlate with gene expression of *FAM65B*, *FAM38A*, and *PRKACA* (Supplemental Fig. S3). In non-malignant samples the DNAm levels at these CpGs were relatively consistent among leukocyte subsets, during hematopoietic differentiation, and between genders (Supplemental Fig. S4). Furthermore, DNAm at these CpGs hardly correlated with chronological age (Supplemental Fig. S5). Notably, the selected CpGs revealed also aberrant DNAm in B-cell lymphoma and MDS, indicating that the aberrant DNAm at these sites is not disease specific (Fig. 2a).

We subsequently designed BA-seq assays for the four AML-associated regions (Supplemental Fig. S6). The reproducibility was validated with three technical replicates and despite variation in sequencing depth the composition of DNAm patterns was highly reproducible in different sequencing runs (Supplemental Fig. S7). We subsequently analyzed 62 blood samples of healthy controls, 48 AML samples at first diagnosis, and 46 follow-up samples, which were classified as MRD positive (*n* = 28) or MRD negative (*n* = 17) according to highly sensitive RT-qPCR detection of the *NPM1* mutation [35]. The BA-seq data validated that AML-associated CpGs were either consistently methylated or unmethylated in the controls, whereas in AML at first diagnosis they display pronounced aberrant DNAm, often even toward the opposite extreme (Fig. 2b). Similar aberrations in DNAm were also observed for the MRD positive samples. Notably, even samples that were classified as MRD negative revealed higher variation in DNAm than the controls, particularly at cg15289427, cg19586199, and cg27630153.

Since not all of the four AML-associated CpGs revealed aberrant DNAm in all AML samples, we combined their DNAm levels into a simple AML-score, ranging from 0 to 1, to discern healthy and AML samples (Fig. 2c). When we applied the AML-score to the Illumina BeadChip data, 99.5% of the control samples (GSE40279, GSE50660, GSE77716; together *n* = 1693) were below 0.125, whereas the vast majority of AML samples was above this threshold (TCGA: 193 of 194; GSE58477: 59 of 62; and GSE62298: 66 of 68), indicating a sensitivity of 98.2%. The AML-score was then tested across five independent studies with clinically diverse datasets (*n* = 275) and revealed aberrant DNAm in 96% of the samples (Supplemental Fig. S8). Furthermore, there was no evidence that the AML-score is higher for samples with adverse cytogenetic or molecular risk score, or that it is associated with specific mutations (Supplemental Fig. S9).

In our BA-seq dataset, the control samples were always below AML-score 0.125, whereas all AML samples tested were above this threshold. The AML-score did not correlate with the *NPM1* mutation burden or blast counts (Supplemental Fig. S10). Furthermore, in the follow-up samples 66% of MRD positive and even 34% of MRD negative samples (*n* = 6) revealed an AML-score higher than 0.125. We specifically screened the six MRD negative samples that revealed positive AML-score for relevant driver mutations. In fact, three of the samples (from two donors) revealed clonal hematopoiesis (*TET2* C1273S, variant allele frequency [VAF] 39%; *IDH2* R140Q, VAF 12%; and *IDH2* R140Q, VAF 30%; Supplemental Table S1). While this might explain some of the residual epigenetic aberrations it did not account for all of these MRD negative samples.

When we analyzed the prognostic value at the first follow-up, the *NPM1* expression was clearly associated with overall survival, event-free survival, and relapse-free survival, whereas this was not significant for the AML-score (Supplemental Fig. S11). Taken together, our AML-score can discern healthy and malignant samples with high specificity and sensitivity. This aberrant DNAm can be observed across many studies and all molecular subsets tested. However, the AML-score does not reliably discriminate MRD positive and negative samples.

### Analysis of DNAm patterns of individual reads in AML

**Fig. 3:**
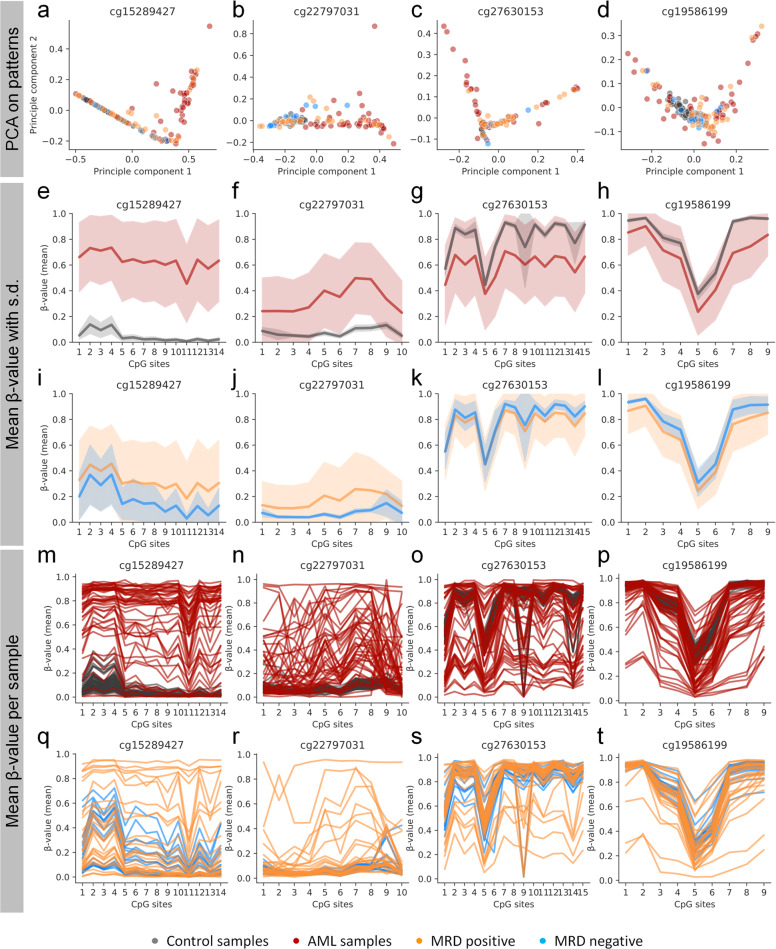
DNAm landscapes at AML-associated regions. **a–d** Principal component analysis (PCA) of the frequency of unique DNAm patterns of BA-seq data for each of the four AML-associated regions. **e–l** Mean *β*-values (±s.d.) of samples (control, AML, MRD positive and MRD negative) at the neighboring CpGs within each AML-associated region. AML-associated aberrations are observed across entire amplicons. **m–t** The DNAm levels of neighboring CpGs are connected for each sample to visualize the variance within the samples.

The selected AML-associated regions comprise several adjacent CpGs (cg15289427: 14 CpGs; cg22797031: 10 CpGs; cg27630153: 15 CpGs; and cg19586199: 9 CpGs) and individual sequencing reads can therefore be translated into a binary sequel of methylated and non-methylated CpGs. To estimate the relationship of DNAm patterns between different donors we performed a principal component analysis (PCA) by using reads of each AML-associated region. Overall, the reads of the controls clustered together while this was not observed for AML samples (Fig. 3a–d). The average DNAm levels at neighboring CpGs demonstrated that entire AML-associated regions revealed aberrant DNAm in AML (Fig. 3e–h). To a lesser degree this was also observed in MRD positive and even in several MRD negative samples (Fig. 3i–l). The maintenance of aberrant DNAm patterns in some of the MRD negative samples was particularly observed in cg15289427 (Supplemental Fig. S12). Notably, within individual AML samples the mean DNAm level of adjacent CpGs was not coherently modified but follow a patient specific pattern, which is in line with our previous results for the age-associated region in the gene phosphodiesterase 4C (*PDE4C*) [37] (Fig. 3m-t). Thus, also the variability of DNAm within short genomic regions can discriminate healthy and malignant samples.

**Fig. 4:**
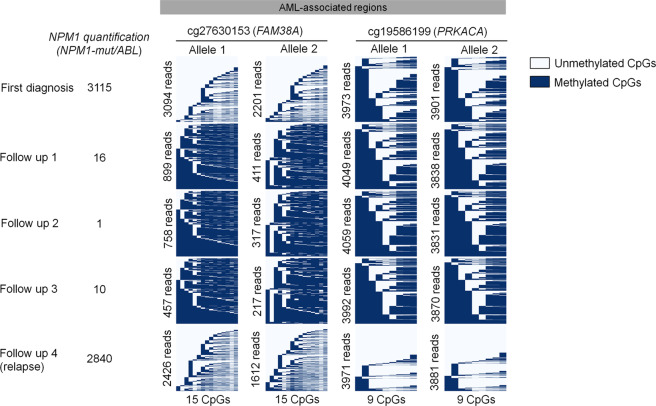
Aberrant DNAm is symmetric between both alleles. The figure depicts DNAm patterns of a patient (#56) with SNPs rs115701567 at chr16:88844998 (cg27630153) and rs917911737 at chr19:14225172 (cg19586199). These SNPs were used to split BA-seq reads of the different alleles. Particularly at first diagnosis the amplicon revealed aberrant DNAm, which normalized during therapy (reflected by lower MRD burden). Upon relapse, both alleles revealed aberrant DNAm patterns. Notably, DNAm patterns were apparently always synchronized between alleles.

Besides imprinting and X-chromosome inactivation, the rest of the genome is thought to be symmetrically methylated at both alleles [38]—possibly due to coherent changes during epigenetic development. However, CpGs of two homologous chromosomes can also exhibit allele-specific DNAm (ASM) [39]. It might be anticipated that particularly the dysregulated DNAm in AML does not affect both alleles symmetrically. AML samples that comprise single nucleotide polymorphisms (SNPs) were utilized to determine DNAm asymmetry in BA-seq amplicons. Notably, we observed that DNAm patterns were always symmetric on both alleles. This is exemplarily depicted for BA-seq reads of patient #56 with SNPs in AML-associated regions of cg27630153 and cg19586199 (Fig. 4): there were significant changes in DNAm patterns at first diagnosis, remission, and relapse, but the DNAm patterns were always almost identical at both alleles.

### Classification of individual DNAm patterns as normal and abnormal

**Fig. 5:**
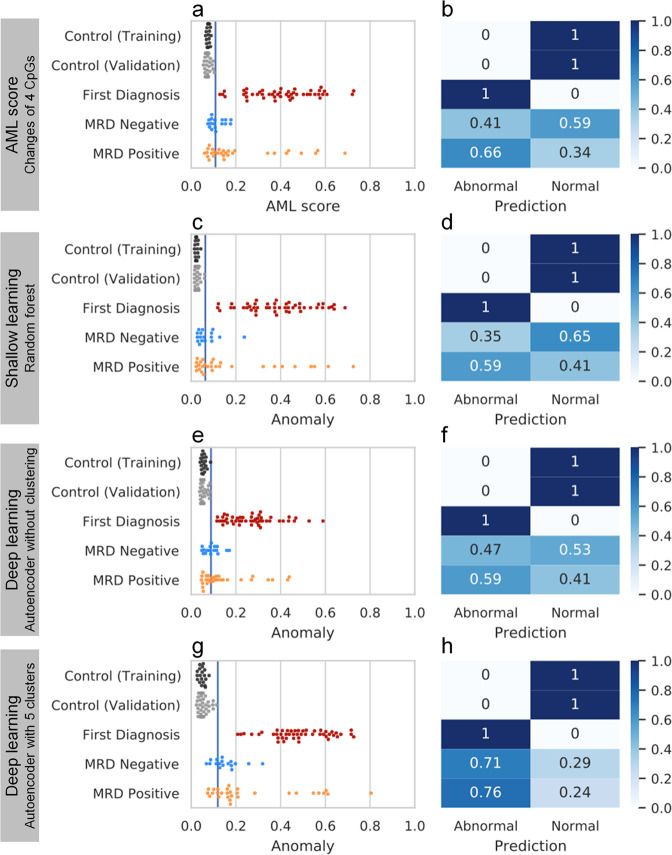
Benchmark of different methods for anomaly detection. The samples of BA-seq were classified as normal or abnormal by four different methods and the percentages are demonstrated in the corresponding confusion matrix: **a,b** AML-score, **c,d** shallow learning by random forest, **e,f** deep learning by autoencoder without clustering, and **g,h** deep learning by autoencoder with clustering. All methods used the same training dataset and the same validation dataset of controls (blue line indicates the threshold of 99% percentile in the training set). All anomaly ratios range from 0 to 1.

Next, we used machine-learning algorithms on the binary DNAm patterns to classify individual BA-seq reads as normal or abnormal. This might facilitate better classification of MRD positive and MRD negative samples than the AML-score, which was based on mean DNAm levels at individual CpGs (Fig. 5a,b). We used shallow learning (Random forest) algorithm to train the model with 6.5 million reads of 43 healthy control samples and 2.6 million reads of 34 first diagnosis samples. For each AML-associated region, random forest uses the DNAm status of each CpG site and builds a decision tree from the DNAm patterns. Hereby, the controls in the validation set revealed a low anomaly score, whereas all AML samples at first diagnosis had a higher score (Fig. 5c,d). Since our MRD samples were from *NPM1* mutated diagnosis samples, we trained the random forest only on the *NPM1*-positive AML first diagnosis samples, but this did not increase the anomaly detection in MRD positive or negative samples (Supplemental Fig. S13).

Alternatively, we used deep learning (autoencoder) either with or without clustering. This method is trained only on the controls and does not require an AML training set. The unsupervised clustering is designed to distinguish various cell types in a sample by their DNAm landscape and enable the autoencoder to learn the pattern with higher specificity (Fig. 5e-h). The prediction results of different methods were subsequently compared with confusion matrixes (Fig. 5b-h). All methods could perfectly separate control samples and AML first diagnosis but had low accuracy in separating MRD positive and MRD negative samples. Autoencoder with clustering could correctly classify 76% of MRD positive samples, but it also classified 71% of MRD negative samples as abnormal.

### Sensitivity of anomaly detection in limiting dilution

**Fig. 6:**
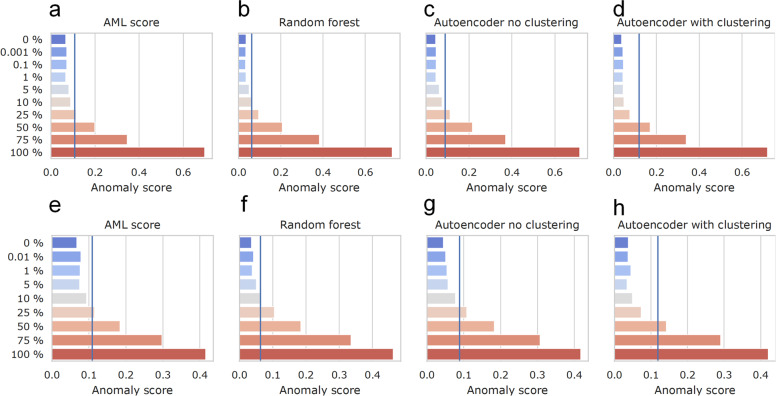
Sensitivity of anomaly detection in limiting dilution. DNA of AML samples from **a–d** bone marrow or **e–h** peripheral blood were mixed with the DNA of a healthy sample in various dilution steps. The four methods for anomaly detection (AML-score, random forest, autoencoder without clustering, and autoencoder with clustering) were compared on these dilutions (blue line indicates the threshold defined in the training set).

The detection of residual *NPM1* mutations with RT-qPCR is highly sensitive (*NPM1*^mut^/ABL < 0.001%), but this approach is not feasible in patients without such mutations. To estimate if MRD analysis could also be performed based on our DNAm patterns we performed a limiting dilution of an independent AML patient (DNA from either bone marrow or peripheral blood) and a control sample (Supplemental Fig. S14). In this dilution experiment, the AML- and anomaly-scores consistently declined with further dilution of both peripheral and bone marrow AML samples until 5% of malignant DNA. The sensitivity of detecting malignant DNAm patterns by using the previously described methods reached around 25% in random forest and autoencoder (Fig. 6). The method was limited by the fact that even healthy blood comprised some reads that were classified abnormal. Furthermore, we tested the algorithms with six leukemic cell lines (HL60, KG1a, TF-1, K562, U937, and SUP-B15). All of them were classified correctly as abnormal, but even in these clonal cell lines DNAm patterns were heterogeneous (Supplemental Fig. S15). Due to this variability of DNAm patterns in control and malignant samples, the threshold to detect residual malignant cells was much higher than with conventional methods for MRD detection.

## Discussion

Acute myeloid leukemia (AML) is a very heterogeneous group of diseases. Despite this heterogeneity, our study demonstrates that targeted DNAm analysis at few genomic regions can very reliably discern healthy and AML samples. It is well known that specific CpGs are more susceptible to aberrant DNAm across various types of cancer, but the underlying mechanism is still unclear [40]. The epigenetic aberrations may be entailed by the frequent AML-associated mutations in epigenetic modifiers, such as *DNMT3A*, *TET2*, *ASXL1, MLL*, and *EZH2*. However, epigenetic dysregulation overall seems to occur independent of the genetic background—the increasing epigenomic plasticity may rather be due other processes, such as aging-associated changes in DNAm or epigenetic drift [40–42]. Either way, the hotspots for AML-specific aberrations in DNAm provide new perspectives, not only for diagnosis, but also for monitoring of therapeutic response and estimation of residual disease burden after treatment. Advantages of using DNAm as a biomarker are the quantitative metric of DNAm levels at single nucleotide resolution, independence of the presence of genomic mutations, and applicability to small volumes of frozen material.

Our targeted analysis showed very high DNAm differences between individual AML samples. Notably, almost all AML samples showed at least at individual AML-associated CpGs higher Δ*β*-values than expected with regard to the relative percentage of blast counts. This result might indicate that the fraction of malignant cells was underestimated by blast counts, or that DNAm changes are also evolved in the non-malignant hematopoietic compartment. The fact that aberrant DNAm did not correlate with blast counts and at least partly remained in MRD negative samples might also be attributed to extrinsic influences by the microenvironment. In fact, several studies demonstrated that changes in the bone marrow can precede the initiation of genetic events associated with neoplasia by creating a pro-malignant state [43]. It is hence conceivable that perturbations in the microenvironment impact on the epigenetic makeup of hematopoiesis.

Furthermore, we demonstrate that the aberrant DNAm is symmetric at homologous chromosomes. It is remarkable how similar the DNAm patterns were on both alleles, given that malignant transformation is usually initiated by heterogenous driver mutations. While many studies described allele-specific methylation (ASM)—which occurs particularly in cancer-associated hypomethylation, disruptive SNPs, and transcription factor binding sites [44]—little is known how the assimilation of DNAm patterns on different alleles is actually mediated. Particularly in plants, paramutation is a well-studied epigenetic phenomenon, in which trans-communication between two different alleles leads to meiotically heritable transcriptional silencing of one of the alleles [45]. This process seems to be mediated via endogenous RNA-silencing pathways and similar interaction might also exist in the mammalian system [46–48]. We speculate that the coherence of DNAm patterns at AML-associated regions is governed by a similar inter-allele epigenetic crosstalk. A better understanding of this process might enable targeting of epigenetic plasticity during malignant transformation.

Many studies analyzed DNAm patterns in primary AML samples, but only little is known how these epigenetic modifications change upon therapy. Notably, even in complete remission, without detection or residual *NPM1* mutations, we observed aberrant DNAm patterns that are hardly detected in normal controls. For therapy-related myelodysplastic syndromes after curative treatment of *NPM1*-mutant AML it has been demonstrated that there can be pre-existing or even newly developed clonal pre-leukemic hematopoiesis, concomitantly with the acquisition of new somatic alterations (such as *DNMT3A*, *IDH1/2* and *TET2* mutations) [49]. Furthermore, we demonstrated clonal hematopoiesis in some samples that were classified as MRD negative. The fraction of samples with clonal hematopoiesis might be higher with a genome wide mutational screening and a more sensitive cutoff than 5% variant reads. Thus, it is conceivable, that aberrant DNAm in our MRD negative samples is due to pre-existing, therapy associated, or newly formed epigenetic aberrations. In the future, it will be important to better understand residual and new occurring genome wide DNAm changes in remission and relapse.

In this study, we have compared different approaches for anomaly detection in BA-seq data. The AML-score is the simplest method to combine the mean DNAm levels at four AML-associated CpGs, but it cannot capture the DNAm changes in a broader landscape and does not take individual reads into account. Furthermore, we used different deep learning algorithms to classify normal and abnormal reads: Random forest utilizes all CpGs for building the decision tree for clustering, but it may not work well when there is no clear cutoff due to the high heterogeneity in AML [50]. Autoencoder was able to take advantage of the massive number of reads for learning and treated all CpGs in a data-driven and pragmatic manner [51]. However, it didn’t perform well with or without unsupervised clustering. The biggest challenge to use DNAm patterns for MRD diagnostics seems to be that even healthy samples had a low percentage of reads that were classified as abnormal. Therefore, DNAm-based monitoring of MRD was not feasible at high sensitivity.

The established methods for MRD diagnostic, such as MFC, PCR, or sequencing approaches, are highly sensitive and can detect down to 0.001% of malignant cells [3, 6]. In contrast, due to some variability in DNAm patterns of control samples the cutoff for our current method is below 5% malignant cells—it therefore does not yet facilitate robust MRD diagnostic. This is also reflected by the finding that MRD monitoring based on RT-qPCR for residual *NPM1* mutation was clearly associated with overall survival, event-free survival, and relapse-free survival, whereas this was not observed for the AML-score. Either way, it is conceivable that in combination with other MRD assays there might be a prognostic benefit, but this would require a systematic larger study. The AML-score may be valuable to support morphological analysis if suitable mutations or surface markers are not available and it may reveal occurrence of clonal pre-leukemic hematopoiesis. The sensitivity and specificity for relapse of AML might be improved with other, or additional, genomic regions. Furthermore, alternative sequencing methods that provide longer reads, such as nanopore sequencing, may enable more reliable distinction between normal and abnormal DNAm patterns. Future insights into the targeting mechanisms that direct AML-associated DNAm changes and of how the epigenetic patterns are assimilated and modified in the course of therapy will provide new perspectives for diagnosis and surveillance of hematopoietic malignancies.

## Supplementary information

Suppl. Fig. S1-S15 and Tables S2-S4

Supplemental Table S1

Supplemental Table S5
