# Supplementary material for: Investigation of measurable residual disease in acute myeloid leukemia by DNA methylation patterns
Source: Leukemia. 2021 Jun 15;36(1):80–9. doi: 10.1038/s41375-021-01316-z (PMC8727289; doi:10.1038/s41375-021-01316-z)
Supplement: Supplementary file 1 — Suppl. Fig. S1-S15 and Tables S2-S4 [file 41375_2021_1316_MOESM1_ESM.pdf]

Supplemental Information

Investigation of Measurable Residual Disease in Acute Myeloid Leukemia by DNA Methylation Patterns

Tanja Božić[1,2,*], Chao-Chung Kuo[1,2,*], Jan Hapala[1,2], Julia Franzen[1,2], Monika Eipel[1,2], Uwe Platzbecker[3], Martin Kirschner[4,5], Fabian Beier[4,5], Edgar Jost[4,5], Christian Thiede[6], and Wolfgang Wagner[1,2,5]

[1] Helmholtz-Institute for Biomedical Engineering, Stem Cell Biology and Cellular Engineering, RWTH Aachen University Medical School, 52074 Aachen, Germany; [2] Institute for Biomedical Engineering – Cell Biology, RWTH Aachen University Medical School, 52074 Aachen, Germany; [3] Department of Hematology, Cellular Therapy and Hemostaseology, Leipzig University Hospital, Leipzig, Germany; [4] Department of Hematology, Oncology, Hemostaseology and Stem Cell Transplantation, Medical School, RWTH Aachen University, 52074 Aachen, Germany; [5] Center for Integrated Oncology Aachen Bonn Cologne Düsseldorf (CIO ABCD); [6] Medical Department I, University Hospital Carl Gustav Carus, TU Dresden, 01307 Dresden, Germany. * These authors contributed equally to this work

Supplemental Figures....................2

Figure S1: Comparisons of DNAm in independent datasets of control and AML samples.................... 2

Figure S2: Association of AML-associated DNAm with blast counts.................... 3

Figure S3: Correlation of DNAm of AML-associated CpGs with gene expression of corresponding genes.................... 4

Figure S4: DNAm at targeted regions is hardly affected by cell counts and gender.................... 5

Figure S5: Correlation of DNAm at targeted CpGs with chronological age.................... 6

Figure S6: Design of bisulfite amplicon sequencing (BA-seq) assays.................... 7

Figure S7: Comparison of the DNAm pattern composition in technical replicates.................... 8

Figure S8: AML-score is positive in independent AML-datasets.................... 8

Figure S9: Association of AML-score with cytogenetic and molecular risk.................... 9

Figure S10: Correlation of AML-score with *NPM1* mutations and blast counts.................... 10

Figure S11: Correlation of *NPM1* expression and AML-score with survival.................... 11

Figure S12: Distribution of DNAm patterns in different samples.................... 12

Figure S13: Anomaly detection by random forest trained on *NPM1* mutated AML samples.................... 13

Figure S14: DNAm pattern composition change in limiting dilutions of AML.................... 14

Figure S15: DNAm patterns of AML-associated regions in leukemia cell lines.................... 15

Supplemental Tables....................16

Table S1: Overview of samples and clinical data of the patients.................... 16

Table S2: Primer list for BA-sequencing.................... 16

Table S3: PCR conditions for BA-sequencing.................... 16

Table S4: Barcode sequences for BA-sequencing.................... 17

Table S5: Overview of 26 hypermethylated and 19 hypomethylated AML-associated CpGs.................... 17

Supplemental Figures

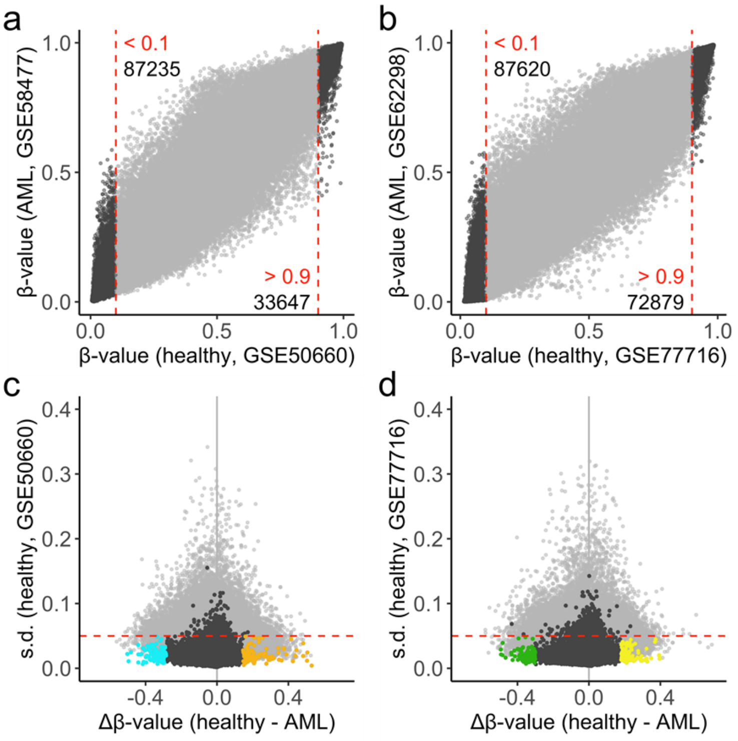

Figure S1: Comparisons of DNAm in independent datasets of control and AML samples.

a,b) Scatter plots of mean DNAm levels (β-values) in Illumina 450K Human BeadChip data of a) GSE50660 (control; n = 464) *versus* GSE58477 (AML; n = 62); and b) and GSE77716 (control; n = 573) *versus* GSE62298 (AML; n = 68). The numbers of CpGs, which were consistently either non-methylated (β-values < 0.1) or methylated (β-values > 0.9) are indicated. Of the 450 000 CpGs represented on the microarray (indicated in grey) about 30% passed this filter criterion (indicated in black). **c,d)** These CpGs were further filtered by low variation in controls (standard deviations [s.d.] < 0.05, red dashed line). Subsequently, the top 100 CpGs with either highest hypermethylation or hypomethylation in AML were selected.

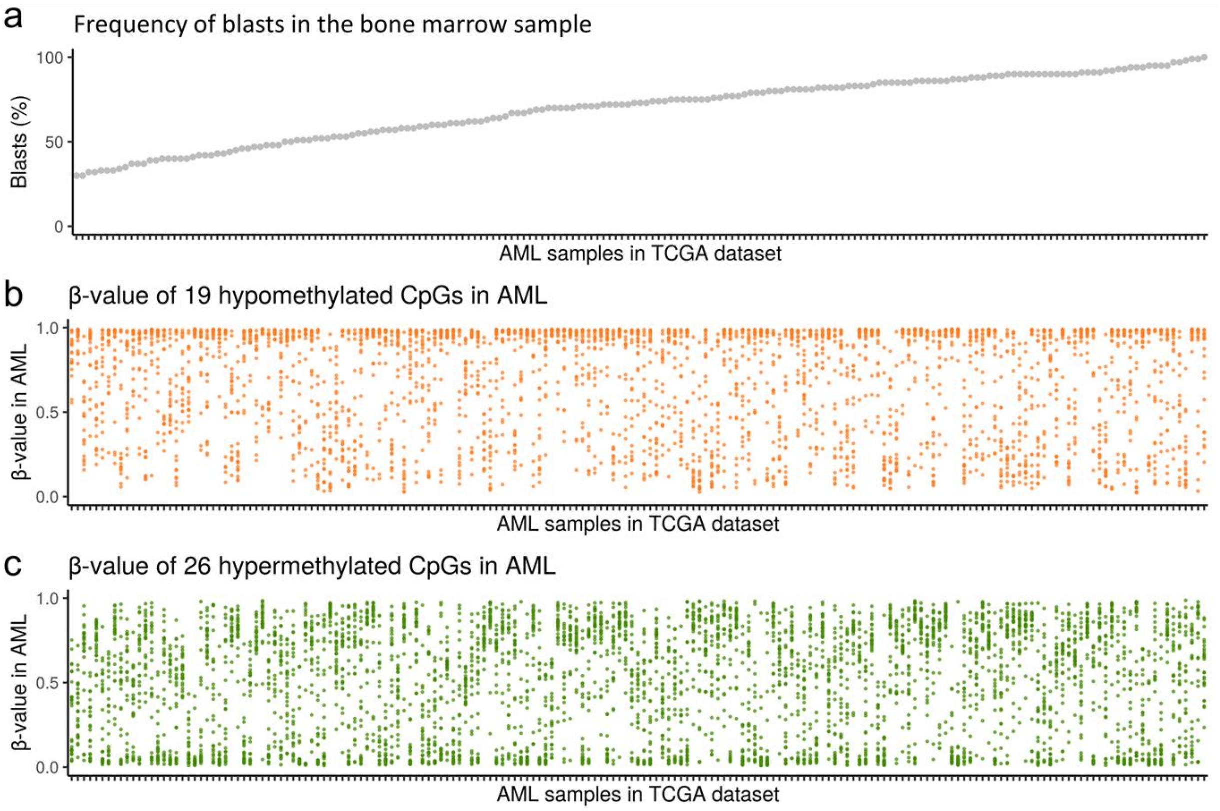

Figure S2: Association of AML-associated DNAm with blast counts.

a) The 194 AML bone marrow samples of the TCGA datasets were sorted by the percentage of blast counts. DNAm levels (β-values) of the **b)** 19 hypomethylated and **c)** 26 hypermethylated CpGs are depicted for each AML sample of the TCGA dataset sorted by the percentage of blast counts. The figure demonstrates that in many AML samples several of the CpGs maintained the normal high or low DNAm, respectively. However, even in AML samples with low blast counts many CpGs revealed distinct aberrant DNAm that indicates that more cells have aberrant DNAm than anticipated by blast counts.

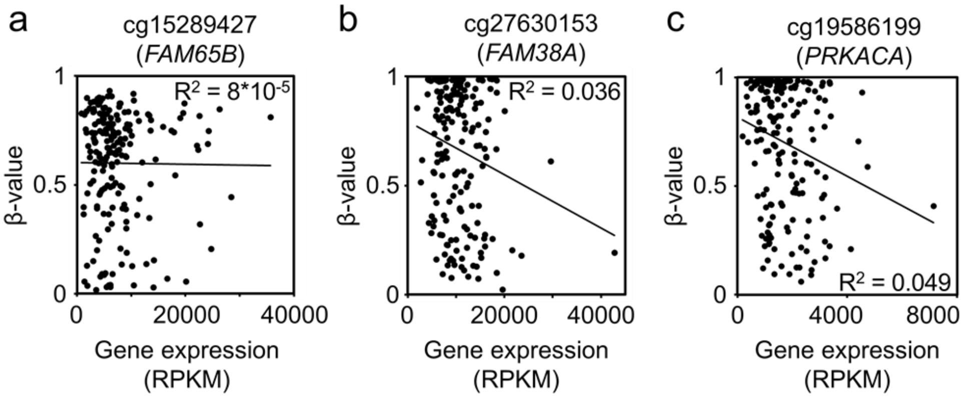

Figure S3: Correlation of DNAm of AML-associated CpGs with gene expression of corresponding genes. DNAm profiles of Illumina 450k from blood samples of 191 AML samples (TCGA) were used to estimate the correlation of DNAm (β-value) with RNA-sequencing data (TCGA) at the three AML-associated CpGs: **(a)** cg15289427 (in 5’ untranslated region of *FAM65B*), **(b)** cg27630153 (in the gene body of *FAM38A*) and **(c)** cg19586199 (in the promotor of *PRKACA*). The CpG site cg22797031 is not related to any gene.

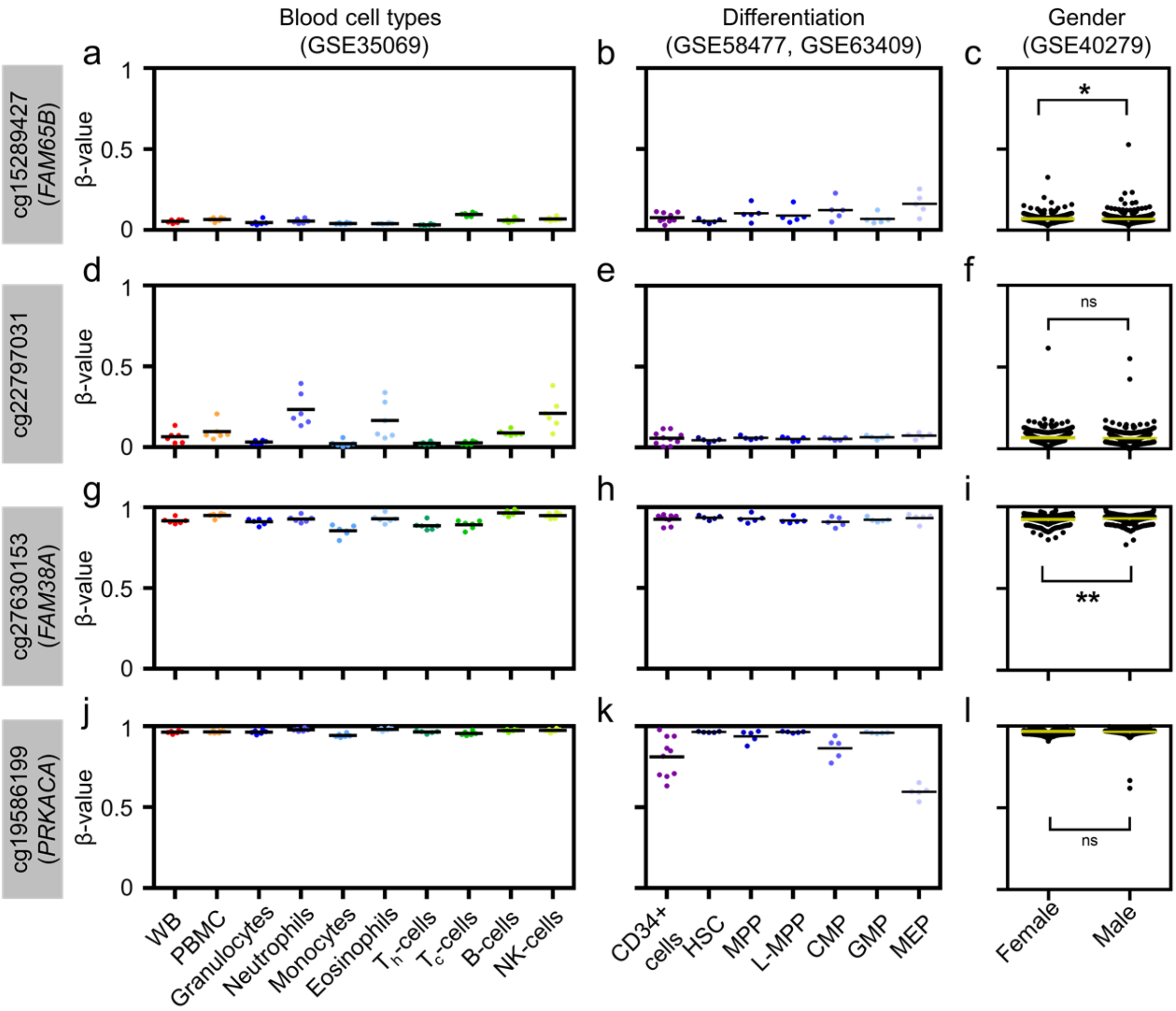

Figure S4: DNAm at targeted regions is hardly affected by cell counts and gender.

DNAm levels (β-values) were analyzed at the four selected AML-associated CpGs: **(a–c)** cg15289427 (*FAM65B*), **(d–f)** cg22797031, **(g–i)** cg27630153 (*FAM38A*), and **(j–l)** cg19586199 (*PRKACA*). Overall, the DNAm levels were very similar in Illumina Human Methylation 450k BeadChip data of sorted leukocyte subsets (GSE35069), while only cg22797031 revealed slightly higher methylation in neutrophils, eosinophils and NK cells. There was also hardly variation in subsets of hematopoietic stem and progenitor cells (GSE63409). Only in $CD34^+$ subsets of bone marrow of healthy volunteers (GSE58477) there was less methylation at cg19586199 than in controls. Furthermore, we analyzed association with gender in the control set with 656 blood samples (GSE40279) and there was only a very moderate, albeit significant, difference at cg15289427 and cg27630153 (Mann-Whitney test. * $P < 0.05$, ** $P < 0.01$, ns – not significant).

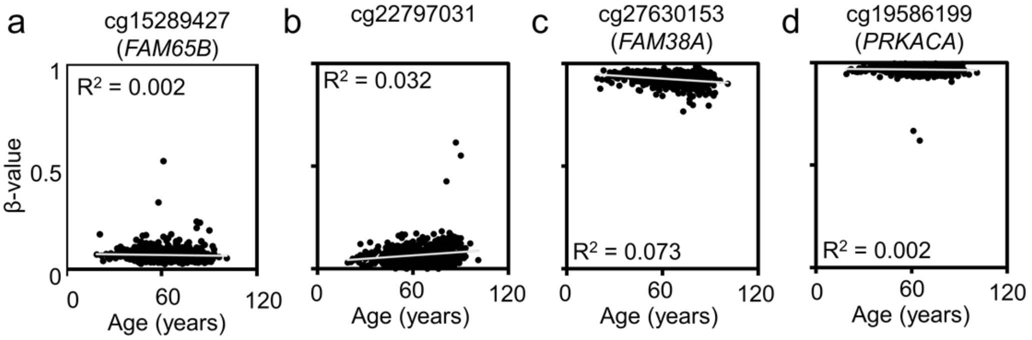

Figure S5: Correlation of DNAm at targeted CpGs with chronological age.

DNAm profiles of blood samples of 656 healthy donors (GSE40279) were used to estimate the correlation of DNAm (β-values) with age at the four AML-associated CpGs: **(a)** cg15289427 (*FAM65B*), **(b)** cg22797031, **(c)** cg27630153 (*FAM38A*), and **(d)** cg19586199 (*PRKACA*). None of the CpGs revealed a clear correlation with chronological age.

>cg15289427 (*FAM65B*), chr6:24910743-24911059 (hg19)

```
CCAACCCCGGGCAATGCTGTGGCCCCTCTAACCTTGGCCCGTTCCTCCACTCCCCAGGGCATCC
                                       1
TTCAGTTCTGCTTGCAAAAAGTGCGCGTGCCCCTTTCGCTCAGCAACTTCCCGACCGTACCCTGT
                       2 3          4              5   6
GGGCTTCAGATGCCCGAACCCACCAGGGTGGTGGAAGCTGAGGACGCTCCAGTTTCAACGGCTGC
              7                             8             9
TTAGCATTCAACGCTCATTCAAGTCGAGGACACCCCTCCCCACACAGACCTCACCGCGTTGTACG
           10           11                           12,13    14
CAAAATCAGAAGCAAAAAAGGAGAAATGAAAAGGTGAACCTACCTAACGACGACGGC
```

>cg22797031, chr1:170630003-170630204 (hg19)

```
TTGATATTAACACGAATGACAAGTGGGTGATTTTCAAGAAGCGCCCGGTCCCTCTAGAGAATGCG
                                         1   2                3
TCCGAATATCAGCGGAGCCGACTGCGTATGCCTCCGGATGCCCATCTATAAACTCTCTTGCTTGT
  4         5     6     7         8
AGCTATTCCTCGCTCCCCAACCATATTGACCATTCACCCGGATAAGGCAATTTCCTCGAAAGGGC
          9                          10
GATCTGA
```

>cg27630153 (*FAM38A*), chr16:88844790-88845210 (hg19)

```
CAGAAAAATAGTGTGCAGATTGGAGCCTGGTAAACAGCCGGCAGCCAAACCCTGCCGGCCCGTCA

GAGGGAGCCTGAAATACTCCCTGTAATTATGCTATTAAAACACACCCGAAAAATTGATCCACCCC

ACTCGGCAAAGGTTCCTGAGACAACGGCGGGGAATCGGGTGGCCACGCACGTCGGCCTAAGGGGC
                        1  2       3         4   5  6
CCTGGCAGCGCCCGGCAGGACAGCCGTCCACTGCACACCTCAGAGCCGGCACCCGGACGGTCAG
        7   8           9                    10     11   12
CGTCCCCATGGGCCCGGCACCAGCAGCTGCCCAGATCCTCACCCGCACACAGGAAGGGTCCCTGC
13            14                          15
AACCCAGCGTCCACCGTGGAGAAACATGAACGTGAGCAAAAATACGGCCTGCGGTGGGTGTGCGA

CCCCACACAGAAGGGCGAGAGGCTCGTGTGC
```

>cg19586199 (*PRKACA*), chr19:14224943-14225293 (hg19)

```
CACCATCGCTGGAGTTGGAAGCCATCACTCAGTCCTGTTCTCAGGGCACCGGCACTACGGTGGCT
                                                1       2
GGGAAGGCTCATGAGACCTGCCGTGTCTGTTCGGCTGTCTGTCCCCAGAACCCTGCCTGCAGGGG
                     3         4
GAGCTAGGGAGCCGTGGGGTGGGAGCAGGAAGAGAAACCCAACCCAGAGGCCACTGGGTGTGGAG
            5
GAGGGACAGATAGGGCCCTCCGAGTTTCTGTAGACGCGGTTGCGCTAAGGGGAGAGCTGCCTTGA
                    6             7 8     9
TAAGACCTCTTGGGCACCCATACAACTGCCAAGGCACAGAAGGTGTTTGGCCGTCTTAGGCATCC

ATAGGATGTTACACGGCACTAGTTCT
```

Figure S6: Design of bisulfite amplicon sequencing (BA-seq) assays.

The DNA sequence of the four AML-associated regions is depicted. Primer binding sites are indicated in gray. CpG sites are highlighted in red and enumerated. CpGs used for the AML-score are underlined.

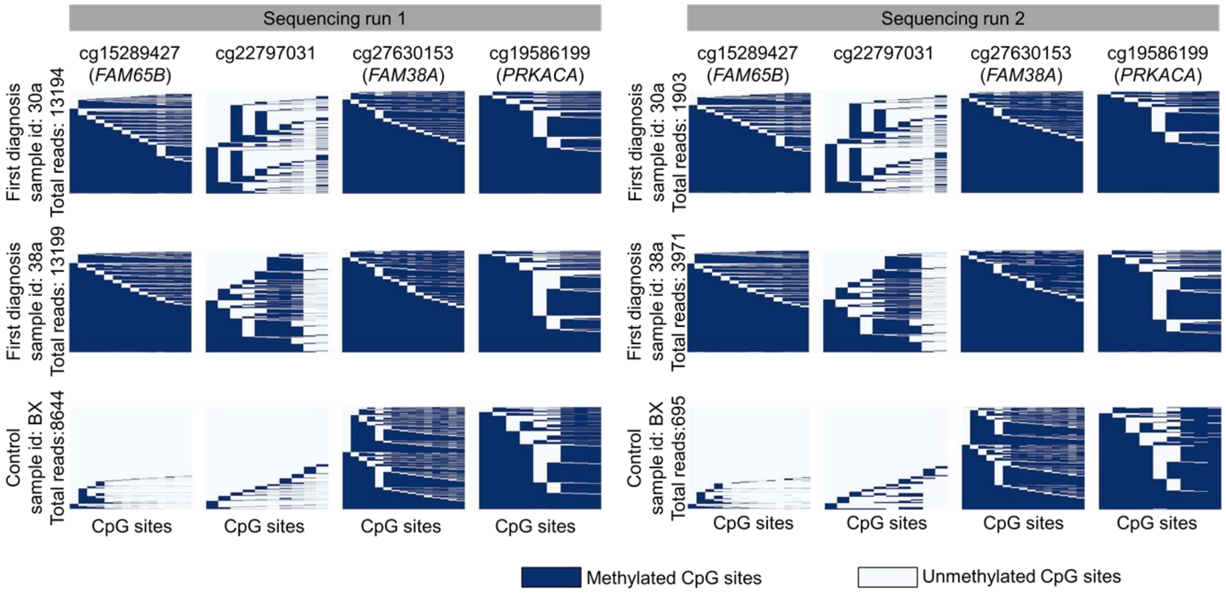

Figure S7: Comparison of the DNAm pattern composition in technical replicates.

The DNAm patterns of the four AML-associated regions were exemplarily depicted for two AML (ID: 30a and 38a) and one control sample (ID: BX) in two independent technical BA-seq replicates. Despite marked differences in the read counts of a sample, the composition of DNAm patterns remained almost identical. Each row indicates one read, while each column represents a CpG sites within the AML-associated region. The patterns in the heatmaps are sorted by the DNAm at consecutive CpGs.

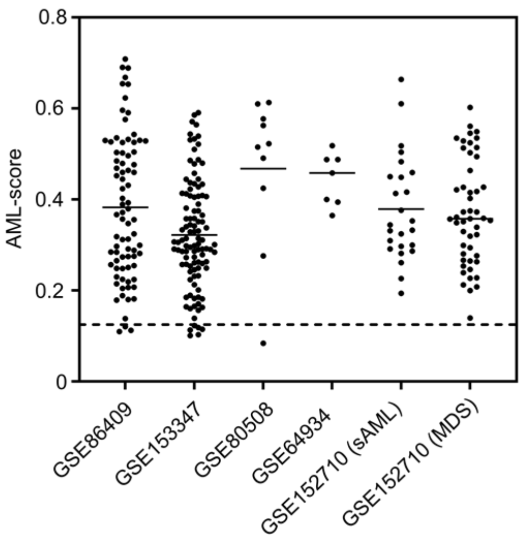

Figure S8: AML-score is positive in independent AML-datasets.

To further evaluate if the AML-score can reliably detect the aberrant DNAm across different types of AML we tested additional datasets: **GSE86409:** 79 elderly AML patients (65-90 years) with different cytogenetic groups that were diagnosed *de novo* (n = 70), secondary (sAML; n = 18) or therapy-related (n = 5); **GSE153347:** 105 longitudinally collected samples of 68 *IDH1/IDH2*-mutant AML patients treated with IDH inhibitors; **GSE80508:** 5 AML positive and 5 AML-M2 negative for t(8;21); **GSE64934:** 3 AML positive and 4 AML negative for *FLT3*-ITD; **GSE152710:** 25 secondary AML patients (sAML); and 49 myelodysplastic syndrome (MDS) patients. The dashed line = threshold of 0.125 (99.5% of controls below this line). The AML-score revealed aberrant DNAm in 96% of the samples.

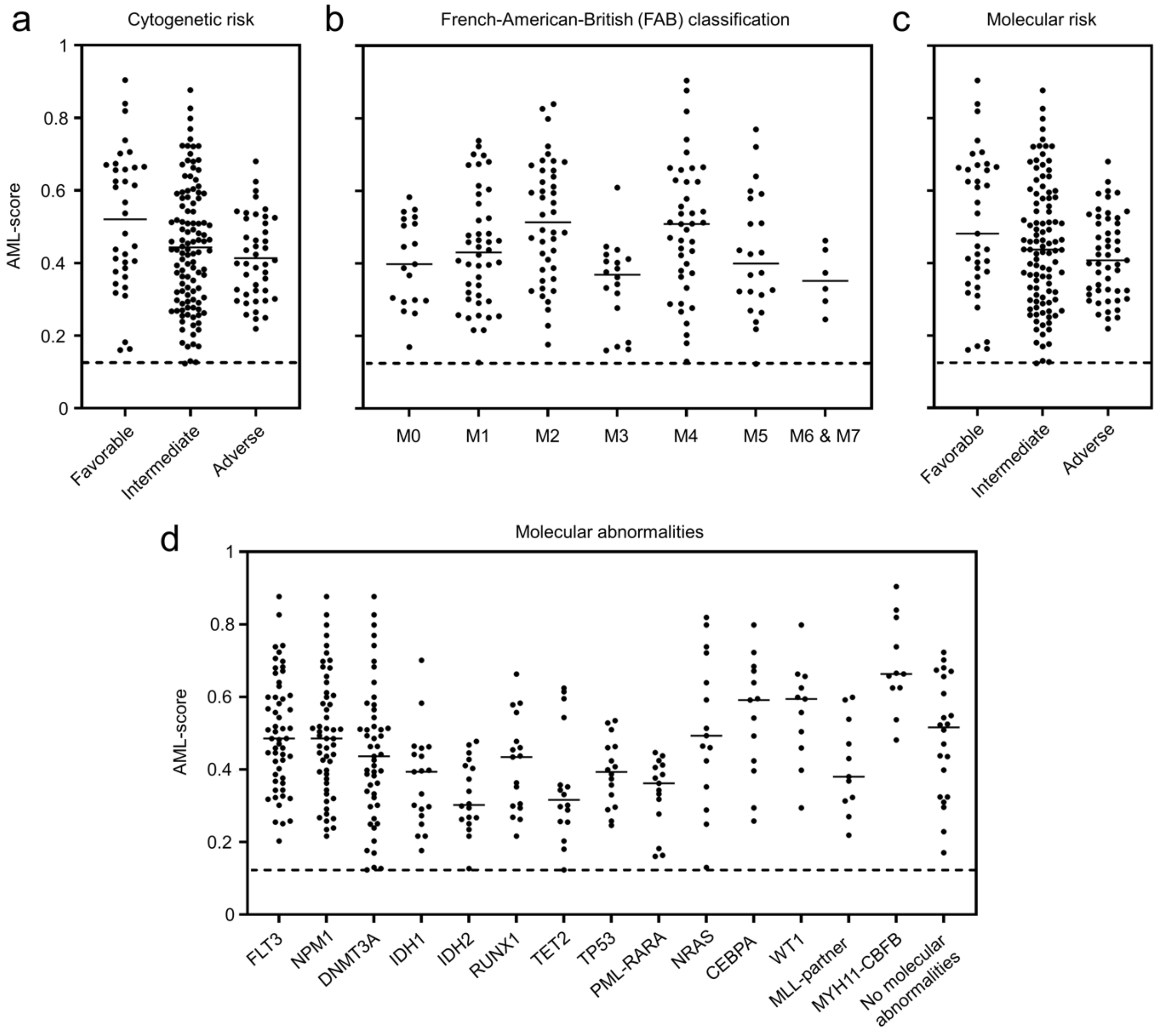

Figure S9: Association of AML-score with cytogenetic and molecular risk.

To gain better insight into how the AML-score is influence by different molecularly defined subsets, we reanalyzed the datasets of The Cancer Genome Atlas (TCGA) by the following categories: **a)** cytogenetic risk scores; **b)** AML-subsets of the French-American-British (FAB) classification; **c)** molecular risk scores; and **d)** molecular mutations or rearrangments (co-ocurrence is not indicated). The dashed line = threshold of 0.125 (99.5% of controls below this line).

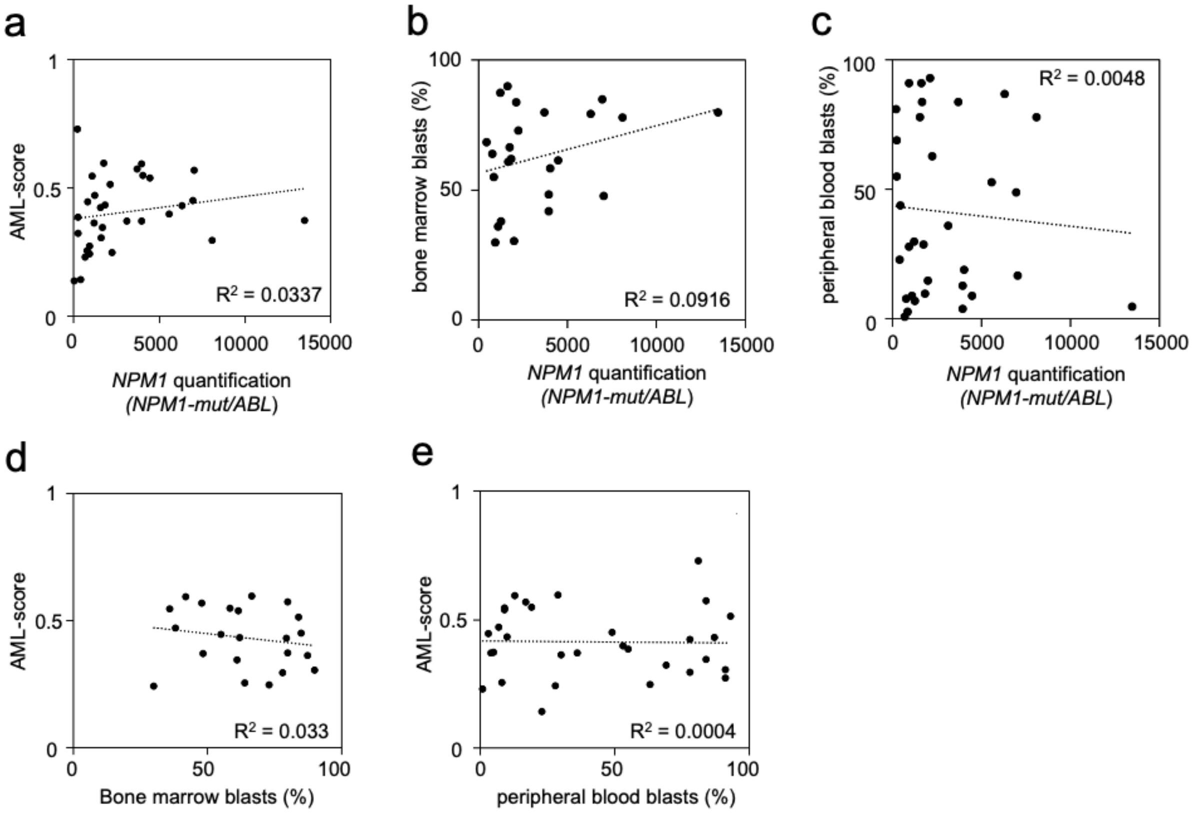

Figure S10: Correlation of AML-score with *NPM1* mutations and blast counts.

Frameshift mutations of nucleophosmin 1 (*NPM1*) in bone marrow of AML samples were monitored by qRT-PCR and adjusted to ABL1 as a reference gene for relative quantification. The *NPM1* quantification did not correlate clearly with **a)** AML-score, **b)** blasts in bone marrow, or **c)** blasts in peripheral blood of 32 AML samples at first diagnosis. Furthermore, the AML-score did not correlate with **d)** blasts in bone marrow, or **e)** blasts in peripheral blood, indicating that it does not provide a reliable measure for the burden of malignant cells.

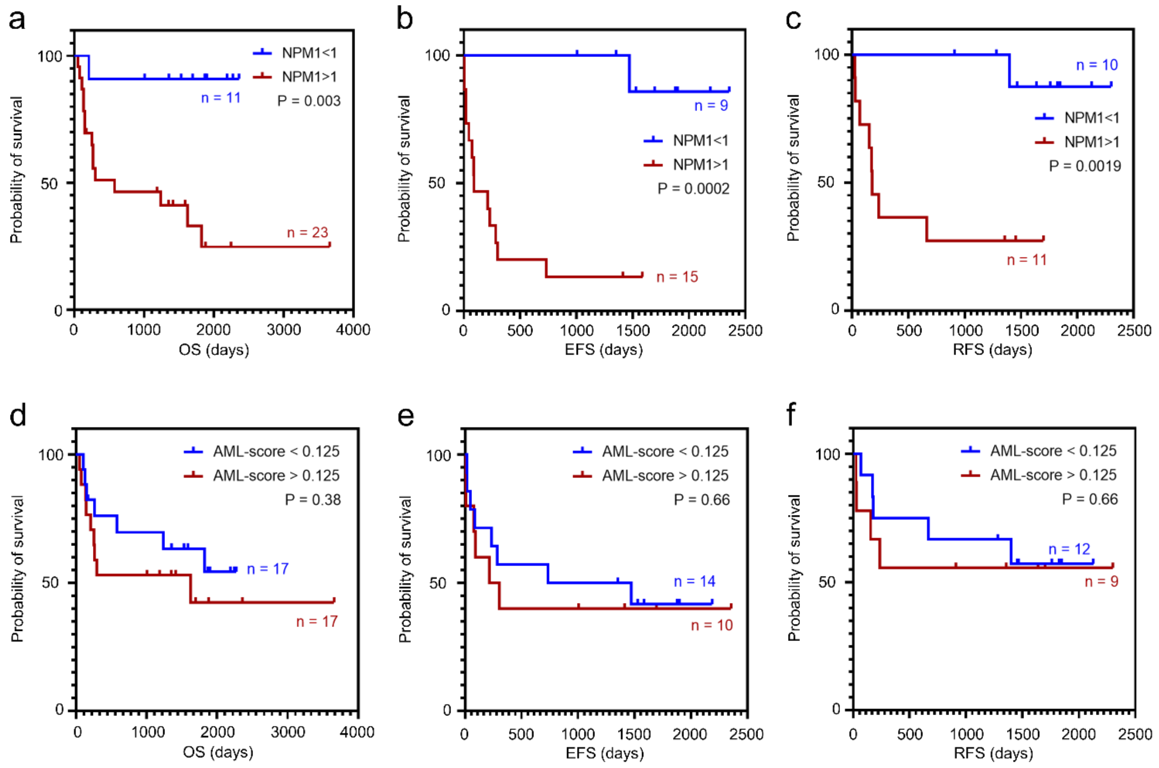

Figure S11: Correlation of *NPM1* expression and AML-score with survival.

AML samples of the first follow-up (n = 34) were stratified by **a-c)** the *NPM1* expression (expression threshold of 1), or **d-f)** the AML-score (threshold of 0.125) and correlated with **a,d)** overall survival (OS); **b,e)** event-free survival (EFS) and **c,f)** relapse-free survival (RFS). The survival time (days) is depicted from the time of *NPM1* or AML-score assessment (first follow-up). Samples with event or relapse before sampling were excluded (therefore the total number of samples differs for OS, EFS, and RFS). The Kaplan-Meier estimate supports the prognostic value of *NPM1* expression, while AML-score did not show significant association. The same non-significant trend was observed when we additionally included six follow-up samples of the AML samples from Aachen with information on OS but unknown MRD status (not depicted, Supplemental Table S1).

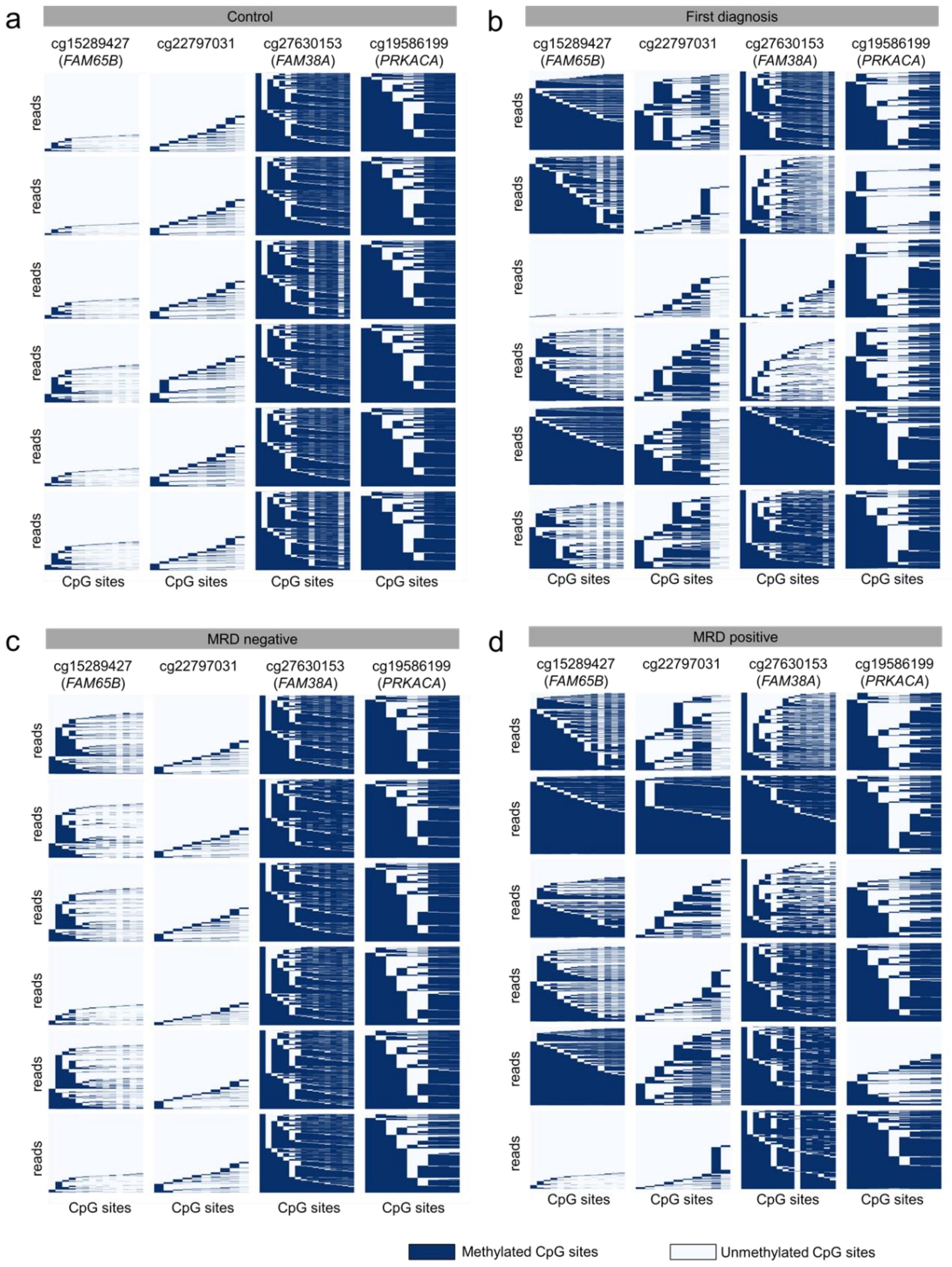

Figure S12: Distribution of DNAm patterns in different samples.

The composition of DNAm patterns of BA-seq reads within the four AML-associated regions is exemplarily depicted for **a)** 5 control, **b)** 5 AML samples at first diagnosis, **c)** 5 MRD negative, and **d)** 5 MRD positive samples. MRD – measurable residual disease. Each row indicates one read, while each column represents a CpG sites within the AML-associated region. The patterns in the heatmaps are sorted by the DNAm at consecutive CpGs.

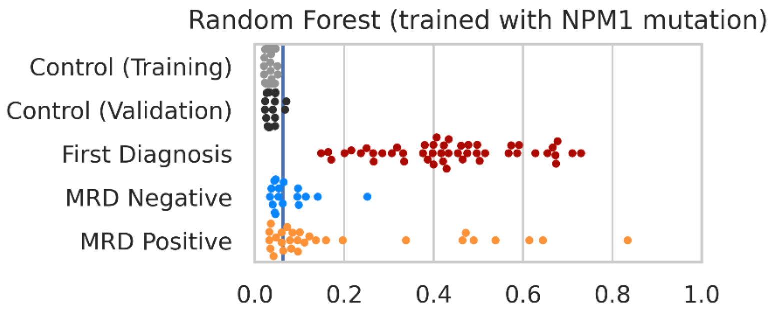

Figure S13: Anomaly detection by random forest trained on *NPM1* mutated AML samples.

The random forest predictor was initially trained on all AML diagnostic samples *versus* controls. Since the MRD samples were from NPM1 mutated patients, we have alternatively retrained the shallow learning algorithm taking only the *NPM1* mutated AML samples and all controls (training and validation) into account. BA-seq data, training and validation procedures are the same as described in Figure 5 (for comparison, blue line indicates the threshold as in Figure 5C). This model could also reliably discern all control and first diagnosis samples, but it did not improve distinction of MRD positive and negative samples.

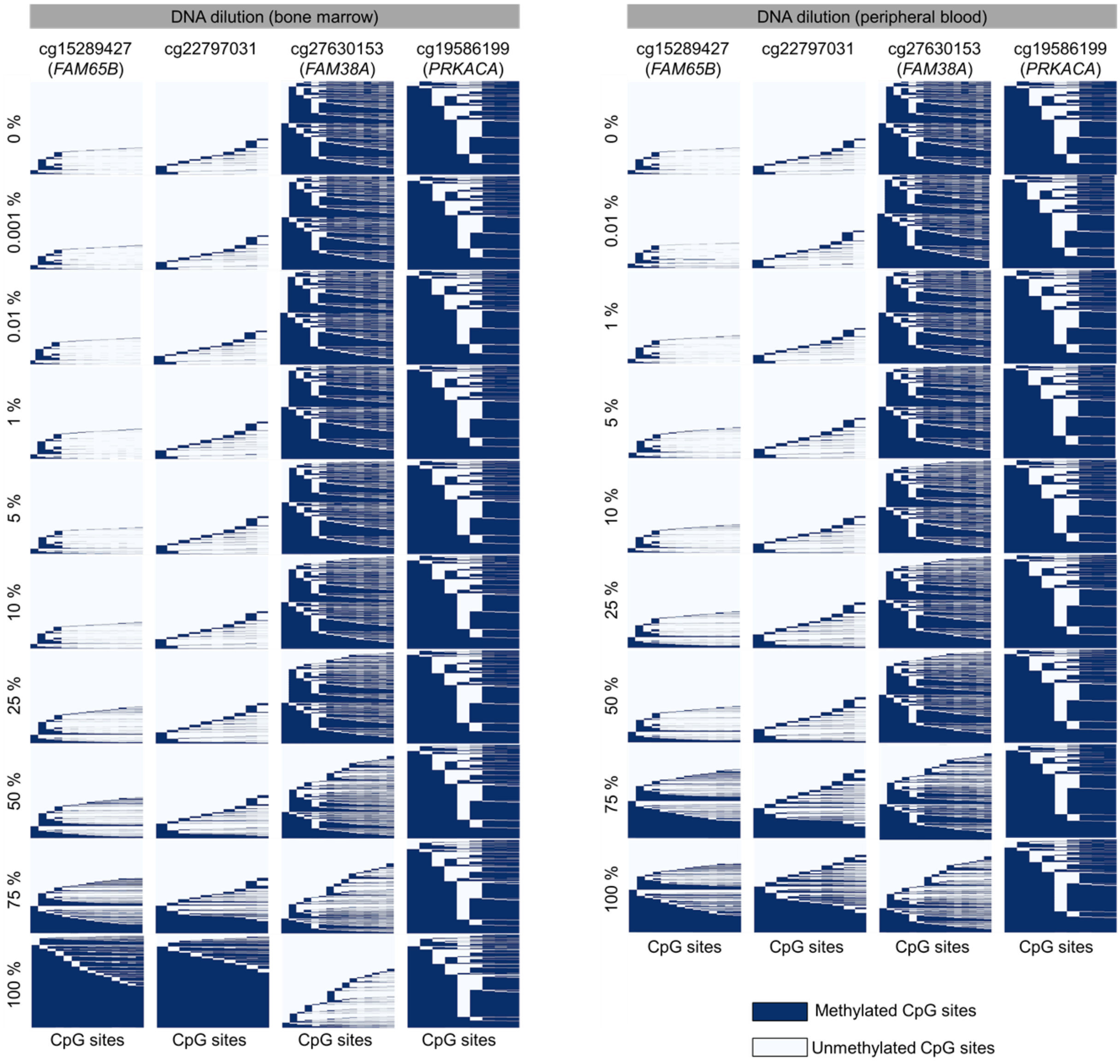

Figure S14: DNAm pattern composition change in limiting dilutions of AML.

DNA of a control sample was mixed with DNA from either bone marrow or peripheral blood of an AML patient at first diagnosis. The dilution steps are indicated (0% = only DNA of the control sample; 100% = only DNA of the AML sample). BA-seq of the four amplicons demonstrates how aberrant DNAm patterns become evident particularly at higher percentages of AML. Each row indicates one read, while each column represents a CpG sites within the AML-associated region. The patterns in the heatmaps are sorted by the DNAm at consecutive CpGs.

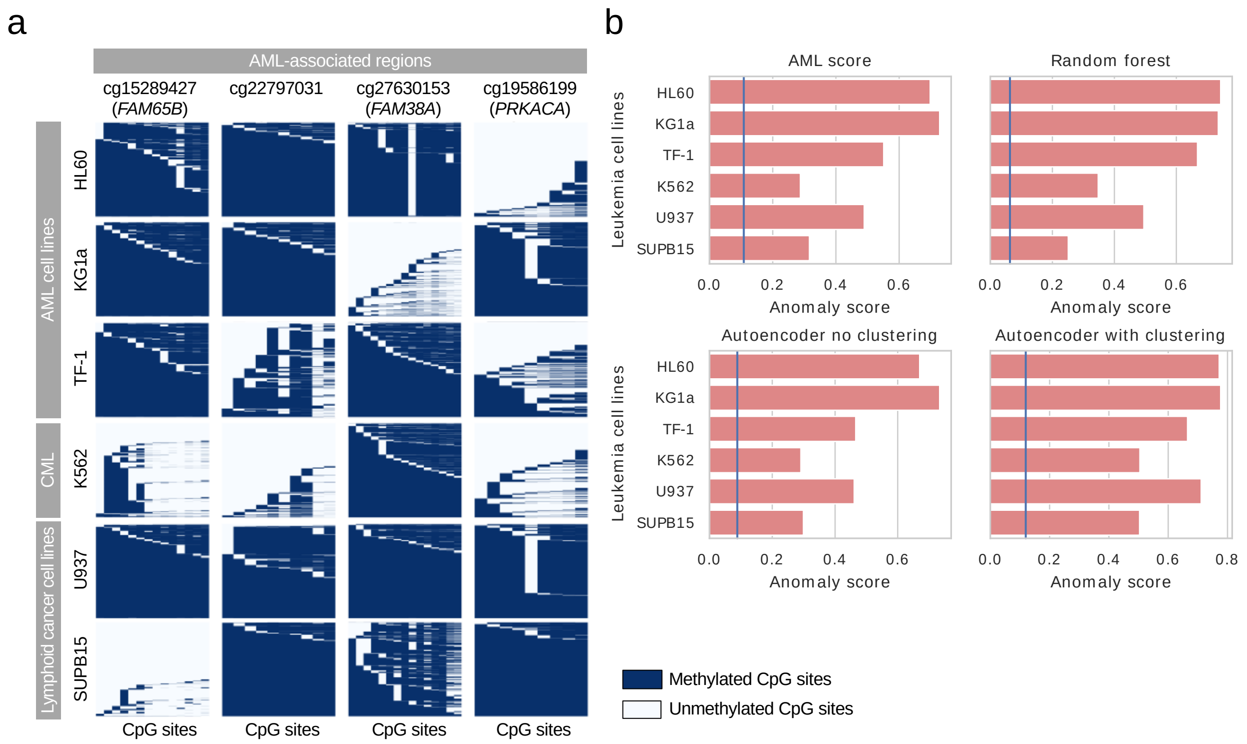

Figure S15: DNAm patterns of AML-associated regions in leukemia cell lines.

a) Six leukemia cell lines were analyzed with BA-seq: HL60 (AML), KG1a (AML), TF-1 (AML), K562 (CML), SUPB15 (ALL), and U937 (Lymphoma). Despite the clonality of the cell lines, the DNAm patterns of AML-associated regions are diverse among AML cell lines and other entities of blood cancer. **b)** The four different methods for anomaly detection were tested on the BA-seq data of leukemia cell lines. Blue lines indicate the 99.5% threshold of healthy control samples in the training set. AML – acute myeloid leukemia; CML – chronic myeloid leukemia; ALL- acute lymphoblastic leukemia.

Supplemental Tables

Table S1: Overview of samples and clinical data of the patients.

This table is provided as separate Excel file.

Table S2: Primer list for BA-sequencing.

Primer	Sequence (5’ to 3’)
**cg15289427 [*FAM65B*]**	
Forward	CTCTTTCCCTACACGACGCTCTTCCGATCTAATTTAGGGTAATGTTGTGGGTTTTTTTAATTT
Reverse	CTGGAGTTCAGACGTGTGCTCTTCCGATCTTCCTCCTTAAATAAATTCACCTTTTCATTTCTC
**cg22797031 [no gene]**	
Forward	CTCTTTCCCTACACGACGCTCTTCCGATCTGATATTAATAAGAATGATAAGTGGGTGATTTTTAAGAAG
Reverse	CTGGAGTTCAGACGTGTGCTCTTCCGATCTAAATCCCCCTTTCCAAAAAATTACCTTATCC
**cg27630153 [*FAM38A*]**	
Forward	CTCTTTCCCTACACGACGCTCTTCCGATCTTTTATTCGGTAAAGGTTTTTGAGAT
Reverse	CTGGAGTTCAGACGTGTGCTCTTCCGATCTCACCAACCTCTCCCCCTTCTATATAAAATC
**cg19586199 [*PRKACA*]**	
Forward	CTCTTTCCCTACACGACGCTCTTCCGATCT TTGGAGTTGGAAGTTATTATTTAGT
Reverse	CTGGAGTTCAGACGTGTGCTCTTCCGATCTCAAAAAATCTTATCAAAACAACTCTC

Green – overhang sequences.

Table S3: PCR conditions for BA-sequencing.

Step	PCR1			PCR2		
	Temp.	Time	Cycles	Temp.	Time	Cycles
Enzyme activation	95 °C	15 min		95 °C	15 min	
Denaturation	94 °C	30 sec		95 °C	30 sec	
Annealing	56 °C	30 sec	35x	60 °C	30 sec	16x
Extension	72 °C	30 sec		72 °C	30 sec	
Final extension	72 °C	10 min		72 °C	10 min	
Hold	4 °C	∞		4 °C	∞	

1.5mM of $MgCl_2$ was used in both PCRs. These same conditions are used for all AML-associated regions.

Table S4: Barcode sequences for BA-sequencing.

Barcoded primer	Sequence (5’ to 3’)
Barcode 1	CAAGCAGAAGACGGCATACGAGATAACGTGATGTGACTGGAGTTCAGACGTGTGCTCTTCCGATCT
Barcode 2	CAAGCAGAAGACGGCATACGAGATAAACATCGGTGACTGGAGTTCAGACGTGTGCTCTTCCGATCT
Barcode 3	CAAGCAGAAGACGGCATACGAGATATGCCTAAGTGACTGGAGTTCAGACGTGTGCTCTTCCGATCT
Barcode 4	CAAGCAGAAGACGGCATACGAGATAGTGGTCAGTGACTGGAGTTCAGACGTGTGCTCTTCCGATCT
Barcode 5	CAAGCAGAAGACGGCATACGAGATCAGATCTGGTGACTGGAGTTCAGACGTGTGCTCTTCCGATCT
Barcode 6	CAAGCAGAAGACGGCATACGAGATCATCAAGTGTGACTGGAGTTCAGACGTGTGCTCTTCCGATCT
Barcode 7	CAAGCAGAAGACGGCATACGAGATCGCTGATCGTGACTGGAGTTCAGACGTGTGCTCTTCCGATCT
Barcode 8	CAAGCAGAAGACGGCATACGAGATCTGTAGCCGTGACTGGAGTTCAGACGTGTGCTCTTCCGATCT
Barcode 9	CAAGCAGAAGACGGCATACGAGATGACTAGTAGTGACTGGAGTTCAGACGTGTGCTCTTCCGATCT
Barcode 10	CAAGCAGAAGACGGCATACGAGATGAATCTGAGTGACTGGAGTTCAGACGTGTGCTCTTCCGATCT
Barcode 11	CAAGCAGAAGACGGCATACGAGATGAGCTGAAGTGACTGGAGTTCAGACGTGTGCTCTTCCGATCT
Barcode 12	CAAGCAGAAGACGGCATACGAGATGATAGACAGTGACTGGAGTTCAGACGTGTGCTCTTCCGATCT
Barcode 13	CAAGCAGAAGACGGCATACGAGATTAGGATGAGTGACTGGAGTTCAGACGTGTGCTCTTCCGATCT
Barcode 14	CAAGCAGAAGACGGCATACGAGATTATCAGCAGTGACTGGAGTTCAGACGTGTGCTCTTCCGATCT
Barcode 15	CAAGCAGAAGACGGCATACGAGATTCCGTCTAGTGACTGGAGTTCAGACGTGTGCTCTTCCGATCT
Barcode 16	CAAGCAGAAGACGGCATACGAGATTCTTCACAGTGACTGGAGTTCAGACGTGTGCTCTTCCGATCT
Barcode 17	CAAGCAGAAGACGGCATACGAGATACCACTGTGTGACTGGAGTTCAGACGTGTGCTCTTCCGATCT
Barcode 18	CAAGCAGAAGACGGCATACGAGATACATTGGCGTGACTGGAGTTCAGACGTGTGCTCTTCCGATCT
Barcode 19	CAAGCAGAAGACGGCATACGAGATACAAGCTAGTGACTGGAGTTCAGACGTGTGCTCTTCCGATCT
Barcode 20	CAAGCAGAAGACGGCATACGAGATAGTACAAGGTGACTGGAGTTCAGACGTGTGCTCTTCCGATCT
Barcode 21	CAAGCAGAAGACGGCATACGAGATCAACCACAGTGACTGGAGTTCAGACGTGTGCTCTTCCGATCT
Barcode 22	CAAGCAGAAGACGGCATACGAGATCAATGGAAGTGACTGGAGTTCAGACGTGTGCTCTTCCGATCT
Barcode 23	CAAGCAGAAGACGGCATACGAGATCACTTCGAGTGACTGGAGTTCAGACGTGTGCTCTTCCGATCT
Barcode 24	CAAGCAGAAGACGGCATACGAGATCAGCGTTAGTGACTGGAGTTCAGACGTGTGCTCTTCCGATCT
Barcode 25	CAAGCAGAAGACGGCATACGAGATGCCACATAGTGACTGGAGTTCAGACGTGTGCTCTTCCGATCT
Barcode 26	CAAGCAGAAGACGGCATACGAGATCCGTGAGAGTGACTGGAGTTCAGACGTGTGCTCTTCCGATCT
Barcode 27	CAAGCAGAAGACGGCATACGAGATGGAGAACAGTGACTGGAGTTCAGACGTGTGCTCTTCCGATCT
Barcode 28	CAAGCAGAAGACGGCATACGAGATGGTGCGAAGTGACTGGAGTTCAGACGTGTGCTCTTCCGATCT
Barcode 29	CAAGCAGAAGACGGCATACGAGATGTACGCAAGTGACTGGAGTTCAGACGTGTGCTCTTCCGATCT
Barcode 30	CAAGCAGAAGACGGCATACGAGATGTCGTAGAGTGACTGGAGTTCAGACGTGTGCTCTTCCGATCT
Barcode 31	CAAGCAGAAGACGGCATACGAGATTTCACGCAGTGACTGGAGTTCAGACGTGTGCTCTTCCGATCT
Barcode 32	CAAGCAGAAGACGGCATACGAGATAAGGTACAGTGACTGGAGTTCAGACGTGTGCTCTTCCGATCT
Barcode 33	CAAGCAGAAGACGGCATACGAGATACACAGAAGTGACTGGAGTTCAGACGTGTGCTCTTCCGATCT
Barcode 34	CAAGCAGAAGACGGCATACGAGATACAGCAGAGTGACTGGAGTTCAGACGTGTGCTCTTCCGATCT
Barcode 35	CAAGCAGAAGACGGCATACGAGATACCTCCAAGTGACTGGAGTTCAGACGTGTGCTCTTCCGATCT
Common reverse primer	AATGATACGGCGACCACCGAGATCTACACTCTTTCCCTACACGACGCTCTTCCGATCT

Blue: Illumina adapter sequence; red: 8-nt unique barcode; green: overhang sequence from PCR1.

Table S5: Overview of 26 hypermethylated and 19 hypomethylated AML-associated CpGs.

This table is provided as separate Excel file.
